# Supportive and non-supportive social experiences following suicide loss: a qualitative study

**DOI:** 10.1186/s12889-024-18545-3

**Published:** 2024-04-27

**Authors:** Franziska Marek, Nathalie Oexle

**Affiliations:** Department of Psychiatry and Psychotherapy II, Ulm University, Ulm/Guenzburg, Germany

**Keywords:** Suicide loss, Suicide bereavement, Grief after suicide, Traumatic grief, Suicide stigma, Social experience, Social support, Interview study

## Abstract

**Background:**

Suicide bereavement entails profound social stressors, including stigma and communication barriers, which can impair social support for suicide loss survivors (SLS). Despite recognized benefits of empathetic interactions, social support, and self-disclosure in mitigating adverse mental health outcomes after suicide loss, we lack a comprehensive understanding of the factors influencing perceived social support among SLS within their broader social environments. To address this gap, our study explores the diverse social experiences of SLS beyond their immediate circles. Specifically, we identify characteristics that define both supportive and non-supportive social experiences of SLS, as well as the facilitators and barriers to social support in the context of suicide bereavement.

**Methods:**

In 2022, we conducted structured online individual interviews with a diverse sample of 18 SLS in Germany. We analyzed these interviews using qualitative content analysis.

**Results:**

We examined the social experiences of SLS across three phases and social contexts: (1) the immediate aftermath of the loss; (2) during bereavement practices; and (3) over time. Our findings show that proactive responses and personalized mourning rituals significantly enhance SLS’ sense of community support, while encounters characterized by avoidance or intrusive curiosity lead to feelings of isolation. Over time, supportive interactions often emerge from peers with similar experiences, promoting openness and shared vulnerability. Conversely, superficial engagement, along with experiences of others depersonalizing and avoiding conversations about the loss, contribute to a sense of marginalization.

**Conclusions:**

Our findings highlight the importance of proactive engagement and open dialogue, calling for societal and communicative shifts toward inclusive and compassionate approaches in addressing suicide loss. This study underscores the need for comprehensive strategies that enhance both suicide and grief literacy and address the taboo and stigma surrounding suicide, ultimately fostering supportive social environments for SLS.

**Supplementary Information:**

The online version contains supplementary material available at 10.1186/s12889-024-18545-3.

## Background

### Social experiences and support after suicide loss

The loss of a close person is a profound and universally shared human experience. Bereavement extends beyond personal grief to encompass a broad spectrum of social dimensions, such as communal mourning rituals [1], expressions of compassion, and social support available to the bereaved [2]. However, bereavement also entails social stressors, including restrictive norms on expressions of grief and stigma toward the deceased and the bereaved, which can impair community support [3]. The social context of bereavement is shaped by various factors, with variations further pronounced by the specific nature of the loss. In Western societies, suicide loss, historically and today, presents unique social challenges for those affected [4, 5], commonly referred to as suicide loss survivors (SLS). Research by Pitman et al. [6] indicates that, compared to those bereaved by sudden natural deaths, SLS receive less support from their social networks. Qualitative findings reveal that SLS frequently encounter awkwardness, gossip, and avoidance within their social environment in response to their loss [7–9]. SLS also often feel judged and shamed by their communities [10], with concerns about causing others discomfort further creating barriers to openly talk about their bereavement with others [11].

Aiming to understand the social challenges and dynamics experienced by SLS, previous research has primarily focused on the stigma associated with suicide [12–14]. Stigma involves complex social dynamics, including labeling, stereotyping, prejudice, and devaluation, which result in the marginalization and discrimination of stigmatized groups [15]. In their study, Sheehan et al. [13] explored the stigma experienced by families bereaved by suicide and found that SLS often confront prejudices of being responsible for the suicide or of having failed to support their family member adequately. The internalization of these negative beliefs (self-stigma) and perceived external stigma are associated with social withdrawal [14] and reluctance among SLS to share their experiences with others [11, 16], isolating them from supportive resources. Moreover, research shows that the societal ‘death taboo’ [17]– a widespread tendency to avoid discussions of death and dying– and the sudden and shocking nature of suicide [9, 17] create communication challenges around suicide bereavement and leave individuals uncertain about how to adequately approach and support SLS [18].

Amidst the silence and stigma of suicide bereavement, empathetic social interactions and support emerge as vital countermeasures to “overcome the walls of stigma and distance” [19]. Research underscores the significance of perceived social support in promoting adaptive coping mechanisms and protecting against mental health challenges associated with suicide bereavement, including depressive symptoms [20–23] and suicidality [20]. Similarly, self-disclosure, the act of sharing personal and emotional narratives, has been identified as a critical aspect of suicide bereavement, acting as a buffer against complicated grief [24, 25] and facilitating loss-related personal growth [16, 26].

Despite these findings, a significant gap persists in our understanding of social support following suicide loss. To address these gaps, it is crucial to recognize the multifaceted nature of social support and the diverse contexts in which it is provided and perceived.

### Identifying research gaps by conceptualizing social support

According to Shumaker and Brownell [27], social support refers to the exchange of resources between individuals, intended to enhance the recipient’s well-being. These resources span emotional (e.g., empathy, love); informational (e.g., advice, guidance); and tangible support (e.g., financial assistance, services), and are provided within a variety of social contexts, ranging from intimate circles like family and friends to community networks and a person’s broader social environment. Adopting a micro-sociological perspective on social support and the interpersonal and communicational dynamics involved, Dyregrov [18] explored the unhelpful social responses experienced by parents bereaved by the sudden death of their child. Her analysis revealed the importance of social experiences beyond immediate family and close friends in shaping perceptions of social support, highlighting “that all encounters between social networks and survivors function as communication and, depending on the meaning created by the survivor, may be interpreted as supportive or as non-supportive” [18]. This is in alignment with Cobb’s conceptualization, which defines social support as “information” [28] that makes an individual feel cared for, valued, and connected to a support network, emphasizing the recipient’s perception.

Despite substantial research on the social challenges and support barriers in suicide bereavement, we lack a comprehensive understanding of the factors influencing perceived social support among SLS within their broader social contexts. Furthermore, existing research has predominantly focused on the deficits and barriers to social support in suicide bereavement, with less attention given to the characteristics of supportive social interactions and strategies to promote them.

### Aim of this study

Extending our focus beyond the immediate social circles of SLS to their broader social environments, this study aims to deepen our understanding of their social experiences and how these influence their perceptions of social support. We refer to ‘social experiences’ and ‘social support experiences’ as the interactions and engagements SLS encounter within their broader social environments, including acquaintances, neighborhoods, the deceased’s social networks, workplace connections, and casual social engagements. Through qualitative interviews, we aim to identify characteristics that define both supportive and non-supportive social experiences of SLS. This approach will allow us to better understand the social dynamics and uncover facilitators and barriers of social support in the context of suicide bereavement, guiding the development of targeted interventions to enhance social support for SLS.

## Methods

### Study design

The present study examines the social support experiences of SLS using online qualitative interviews conducted as part of a mixed-methods study investigating the determinants of social support after suicide loss (DE-LOSS). Following the Consolidated Criteria for Reporting Qualitative Research (COREQ) [29], this section details our methodological approach and its implementation. Additional information on funding, data availability, ethics approval, and consent for participation is provided in the final section of this publication.

### Inclusion criteria and recruitment

Eligibility for participation was determined by the following criteria: being at least 18 years of age; fluency in German; having experienced the loss of a close person to suicide (defined as ‘someone who was important to you and your life’); having been at least 14 years of age at the time of the loss; and having experienced significant emotional distress due to the loss. Loss-related emotional distress was assessed by asking SLS to indicate how distressed they felt by the loss at the time of their greatest distress. Eligibility was determined by a score greater than 3 on a scale ranging from 1 (not at all) to 5 (extremely). This study was designed to examine adult and late adolescent social and support experiences, excluding losses that occurred before the age of 14. The threshold for significant emotional distress was set to identify individuals who were perceived to be deeply affected by grief, warranting support and compassion.

Recruitment took place in Germany from January to March 2022, utilizing regional newspaper ads, emails, and flyers directed at SLS support groups. Recruitment materials outlined the study’s purpose, participation process, compensation details, and contact information. Prospective participants contacted us via telephone, email, or an online form, leading to initial telephone screening interviews. These interviews served to introduce the study, determine eligibility, and address any questions or concerns. Additionally, they provided an opportunity for participants and the researcher to familiarize themselves, fostering a trusting environment for the subsequent online interviews. Participants received a €30 allowance and information about support services, including details of support groups, counseling services, and mental health resources aimed at addressing mental health concerns, grief and suicide loss.

### Participants

From the 106 individuals expressing interest, we aimed for high variation in our sample, considering gender, age, relationship to the deceased, and previous engagement with formal support services. Based on our team’s previous experience of achieving data saturation in qualitative interviews, 18 SLS^1^ were selected and invited for an interview. Those not selected were pre-registered for a subsequent quantitative survey as part of DE-LOSS. Our sample is gender-balanced (9 women, 9 men), aged between 23 and 64 years, with the age at loss ranging from 14 to 63 years. The majority had experienced a single suicide loss, with one individual reporting three such losses. Detailed demographic and loss-related characteristics of participants are available in Table 1.

**Table 1:** Participant characteristics

Participant code	Gender (age)	Relationship of deceased to SLS (deceased’s age at time of death)	Age at loss	Years since loss
P1	f (38)	grandfather (83)	14	25
P2	f (37)	uncle (42) godfather (43) father (70)	19 22 36	19 16 1
P3	f (23)	brother (23)	20	3
P4	m (39)	brother (28)	22	17
P5	m (23)	female partner (22)	22	1
P6	f (33)	father (66)	31	2
P7	f (35)	mother (51)	32	3
P8	m (39)	male friend (36)	38	1
P9	f (55)	father (77)	42	13
P10	f (45)	husband (51)	45	½
P11	f (48)	son (16)	47	2
P12	f (54)	son (22)	51	3
P13	m (60)	daughter (15)	54	7
P14	m (59)	female partner (53)	55	4
P15	m (57)	brother (53)	56	1
P16	m (60)	son (23)	57	3
P17	m (59)	son (26)	58	1
P18	m (64)	wife (61)	63	1

### Data collection

We collected data through problem-centered [31], online individual interviews, guided by a structured approach that included a demographic questionnaire, an interview guide, a recording device, and postscripts. This method prioritizes flexibility during interviews, allowing for subjective relevance and unexpected topics while ensuring thematic consistency and comparability across interviews. The interview guide, developed by the research team through an extensive literature review and conceptual examination of social support, covered various themes related to social support, including family coping and support to broader social and formal support experiences. It underwent review by academic peers and a participatory advisory board consisting of SLS and experts in postvention and suicide bereavement. The focus of this publication primarily centers on narratives pertaining to the interview guide section ‘SLS’ social experiences within their broader social environments,’ available as supplementary material 1.

We conducted the interviews between March and May 2022. The interview sessions lasted between 41 and 93 minutes  and were audio-recorded. Following each interview, the interviewer (FM) compiled a postscript, documenting personal reflections and notable thematic and conversational aspects.

Suicide loss is often accompanied by intense grief reactions and trauma symptoms, exacerbated by factors such as witnessing the suicide and discovering the body [4]. Moreover, SLS express a desire to discuss their loss experience but may hesitate to do so out of concern for others [7, 11]. To ensure a sensitive and open narrative, we encouraged participants at the start of the interviews to respond in the way they felt most comfortable. They were given the opportunity to indicate any topics they preferred not to discuss and were encouraged to request breaks or end the interview if needed. In some cases, the interviewer redirected the conversation, particularly when participants seemed overwhelmed or unable to move past highly distressing experiences. These participants were reminded of the information about support services that was part of the participation materials and were encouraged to contact study staff for further support.

### Analysis

Audio recordings were transcribed verbatim and anonymized, with personal and potentially identifying information replaced by abstract placeholders. The analysis, conducted by a two-person team (FM, NO), employed qualitative content analysis as outlined by Kuckartz and Rädiker [32], integrating systematic coding with computer-assisted analysis (MAXQDA 2022) and triangulation. Initial steps involved thorough and reflective engagement with the data, addressing personal biases, and ensuring the research team’s familiarity with the narratives of SLS. FM compiled comprehensive case summaries for each interview, providing the basis for further analysis. Subsequently, FM proceeded with the coding process by establishing a structured code system that integrated both deductively derived themes from the interview guide and inductively generated themes that emerged from the data. Preliminary code definitions were documented in memos to establish a shared understanding of the coding system within the research team.

Building upon the initial coding phase, FM and NO engaged in an iterative process of applying and refining the code system, incorporating additional themes as they emerged from the data. This ongoing refinement, supported by continuous team dialogue, ensured the coding system accurately reflected the depth and breadth of the data. The evolving code system was documented, with code memos detailing the criteria for code application and including exemplar quotes to illustrate each code’s relevance. The code system and code memos were presented and discussed at a qualitative research workshop and no further changes were made.

In the final stage of analysis, FM synthesized the coded data to address the analytical focus. This entailed selecting significant codes, conducting in-depth analysis and interpretation of these codes. This process was grounded in a collaborative effort, with interpretations validated through internal discussion with the research team and external validation at research workshops involving academic peers and the participatory advisory board.

We translated the original interview quotes for the results section through a collaborative and iterative process, involving team members (FM, NO) and an external bilingual expert. The translation aimed to preserve the semantic integrity of the source material, focusing on accuracy and faithful representation for an English-speaking audience, rather than literal equivalence.

## Results

The social experiences of SLS unfold across three distinct phases and contexts: (1) social experiences in the immediate aftermath of the loss, covering approximately the first two weeks and leading up to the funeral; (2) social experiences during shared bereavement practices; and (3) ongoing loss-related social experiences in their daily lives up to the present.

### Initial reactions

#### Acts of kindness and community solidarity

Following their loss, SLS reported a spectrum of immediate and compassionate responses from their social networks. Acts of kindness, such as delivering meals, sending condolences through letters and cards, and monetary donations, were highlighted by participants. These gestures came from various sources, including neighbors, colleagues, community leaders, and anonymous individuals, underscoring a collective effort to support the bereaved. Participants expressed gratitude for the time, attention, and care shown by these individuals, especially considering the absence of a close personal relationship:

*P3: “For example, our neighbor baked us a cake. It’s little things like these that let you know someone’s made time for you. Although we don’t talk much, except for a bit of small talk now and then. But he made some time and just wanted to do something for us.”*

Similarly, another participant had received an anonymous donation, reflecting the solidarity within the extended community:

*P17: “One thing that really touched us was an envelope we received from the mayor which contained 500 euros. It said that some people, it didn’t go into detail who, had organized a fundraiser for our son, with the intention that the money would be used for [name of deceased]. And that’s something that made us wonder: ‘Who did this? Who cares so much as to organize a fundraiser for him?’”*

#### Social discomfort and avoidance

During the initial bereavement phase, interactions often involved noticeable discomfort and avoidance. SLS described encounters where individuals seemed overwhelmed, resulting in speechlessness and avoidant behavior. In these situations, some SLS felt compelled to alleviate the discomfort of others, despite their own need for compassion and support:

*P10: “I often found myself having to ease their insecurities (…). It made me feel like I had to lead the conversation, like I had to manage it. (…) Some neighbors came over to my house because that’s what you do, you offer your condolences, but they didn’t really know what to say, and I was also completely overwhelmed. Often, I felt relieved when they left.”*

Avoidance extended to digital communications, with a noticeable drop in messages on social media:

*P7: “There was this abrupt stop, and suddenly there were no messages at all. No matter which channel. Except for maybe a couple of contacts. And apart from that, it was quiet for a few weeks.”*

Experiences of social avoidance led some to feel like subjects of community gossip, contributing to feelings of isolation. Especially in smaller communities, gossip and speculation added to the sense of being scrutinized rather than supported:

*P7: “We live in a small village, and there’s been some gossip (…) about there having been definite difficulties in the family, that something wasn’t quite right.”*

*P3: “Yes, I noticed that my classmates were talking about it and wondering, ‘Oh, was it a suicide or not?’ None of them ever asked me.”*

Despite their emotional distress, most participants showed empathy toward others. They attributed the communication gap to the widespread shock and lack of social norms for discussing suicide, which they described as “incomprehensible” and “taboo” (P10).

Several SLS described encountering intrusive forms of communication, including inquiries about sensitive topics such as the location of the death or the deceased’s medical condition. One participant mentioned being able to recognize the intentions behind peculiar questions, distinguishing between curiosity-driven and genuinely supportive expressions of sympathy. Another participant expressed discomfort and embarrassment due to the inappropriate nature of these conversations, particularly in public settings:

*P1: “They asked me, right in the middle of the street, quite casually, ‘That’s terrible, but was it because of his prostate cancer?’ And I just thought: ‘Prostate? What’s that?’ At 14 years old, not a clue.”*

### Bereavement practices

#### Personalized practices and collective mourning

Participants noted the profound impact funerals, memorials, and commemorative events had in honoring their lost loved ones and fostering supportive social interactions. Participants valued the large and often unexpected turnout at these gatherings, including colleagues, distant acquaintances, and the deceased’s friends. Recognizing the effort many had made to attend, participants emphasized the communal support they felt through the presence of diverse groups. One participant described honoring their deceased son with a personalized service at home:

*P17: “Just before the cremation, we brought his coffin here to our property. We set it up in front of his little house in the garden, inside a small tent equipped with a cooling device, as the weather was quite warm. We then invited all his friends, relatives, and anyone who could make it, and many of his friends were there. It allowed us a real chance to say goodbye. (…) Some found it a bit odd at first, doing this at home in the garden. But afterwards, everyone thought it was wonderful, they were totally impressed, saying, ‘It was such a great idea and so beautiful to be able to say goodbye in this way, in familiar surroundings.’”*

These informal gatherings fostered collective mourning and deepened connections with others through intimate conversations and the sharing of memories:

*P16: “After the funeral we invited everyone who wanted to come to our garden. (…) We sat together, sharing fond memories of him. It was somewhat unconventional. Not the typical funeral reception, but rather, how should I put it? A very relaxed gathering. Laughing as we shared various anecdotes about him. Truly, it was the best thing we could have done. Because afterwards, you’re left to fill the void. Had we not done that, we would have fallen into a pit.”*

Participants appreciated these personalized settings for their flexibility and the comforting space they offered for grieving. From their perspectives, as opposed to the formality of traditional funeral services, less formal settings enabled them to navigate social interactions more naturally:

*P16: “Well, it’s about being at home, having that anchor point somehow. (…) And, yes, having the possibility at any time, since many were there, to quickly withdraw if one needed to. The freedom to move among the different groups that had formed.”*

Another participant emphasized how delegating the funeral’s organization enabled him to be more receptive to the condolences shared by attendees:

*P14: “And that was really, really important to me to truly take it all in (…).”*

Participants also engaged in remembrance practices beyond funeral services, recognizing the vital role of nurturing connections with the deceased’s social network. These practices included visits from the deceased’s school class on their graduation day, memorial gatherings with the deceased’s coworkers, and intimate gatherings held at the deceased’s home. Additionally, participants emphasized the meaningfulness of gestures such as distributing the deceased’s belongings among their friends. In one case, friends of a participant’s deceased son established a commemorative association in his honor, which aimed to educate adolescents about mental health and support-seeking. This initiative, viewed by the participant as a form of “grief therapy” (P12) for the deceased’s friends, was cherished as a means to maintain connections with them.

#### Mourning and social expectations

Some SLS faced difficulties aligning their personal grieving process with communal mourning events, feeling pressured by societal expectations to grieve openly and expressively. One participant shared that he found the anticipated expressions of sympathy and the prospect of engaging with others overwhelming:

*P8: “I have to say, I just kept to myself because I didn’t want to talk to those I knew, like his friends or our circle of friends and his coworkers. I knew they would just say, ‘Hey, I’m so sorry, keep your chin up.’ And I really wasn’t in the mood for that kind of empty consolation, that pointless sighing.”*

Conversely, another participant experienced discomfort due to a lack of social engagement during the funeral. She explained how the service was overshadowed by the profound shock of her husband’s suicide, resulting in a stifling atmosphere that inhibited meaningful dialogue:

*P10: “There was also just uncertainty everywhere.”*

Despite their differences, both narratives emphasize the challenges SLS face in finding balance between their personal needs– ranging from solitude and withdrawal to seeking social interaction– and the varying degrees of social engagement observed at mourning events, which can be perceived as either overwhelming or insufficient.

The COVID-19 pandemic posed additional challenges by limiting communal support opportunities and exacerbating feelings of isolation during crucial moments of grief:

*P18: “We couldn’t do anything after the funeral, it wasn’t allowed because of the pandemic. Everything was closed, we couldn’t have gone anywhere.”*

### Social experiences over time

#### Shared experiences and supportive bonds

Participants valued connections with others who had experienced similar losses or significant challenges. These connections, ranging from neighbors and coworkers to fleeting encounters that developed into lasting relationships, provided SLS with a sense of unique depth of understanding and support. In one case, a participant described a chance meeting with another SLS that led to what they called a “revelation” (P9) of mutual understanding:

*P9: “It was a stroke of luck, but it happened two years later. It was just a good, understanding conversation. It was helpful. Meeting someone who had been through the same thing, though not losing a father, but someone else. I must say, it was a good experience. (…) And it was like a revelation. It was unbelievable.”*

Similarly, a participant recounted forming an instant bond with a mother who had lost her son at an age similar to her own child’s, highlighting a profound and unspoken understanding between them:

*P11: “With her, yes, we didn’t need to talk much. It was simply an understanding, an immediate understanding, a blind understanding, yes.”*

Shared experiences fostered social connections in which SLS felt acknowledged, accepted, and genuinely supported. The shared experience of loss, whether sudden or due to the passing of a partner or parent, fostered a supportive environment where explanations were unnecessary, reducing the pressure to explain their grief:

*P8: “I didn’t need to say anything.”*

For instance, a participant noted how his supervisor, also grieving a partner’s loss, demonstrated compassion and understanding, allowing him space to grieve without work pressures. SLS also had meaningful conversations with others who faced challenges like depression, infertility, or parental illness. These shared experiences led to open discussions, emotional connections, and bonds of solidarity:

*P3: “A few months later, [a former friend’s] father suffered a brain aneurysm or something. Then, at some point, we got together. We said, ‘Hey, we both have parents who are going through a tough time.’ And yes, we ended up distracting each other.”*

*P16: “And to suddenly hear from people who would have liked to have children but couldn’t. (…) So, yes, that was also something that really helped us. (…) Yes, it somehow makes you more open.”*

#### Avoidance and insensitivity

Participants expressed distress over interactions where their grief was met with silence, avoidance, or insensitivity. Some participants felt upset and isolated, perceiving their loss as being marginalized within their social environment. These perceptions stemmed from a lack of inquiries about their emotional state, abrupt shifts in conversation, and a general avoidance of topics related to the loss, sometimes resulting in the deceased being erased from conversations with others:

*P3: “No one ever asked me how I was doing. (…) And when I brought it up myself, there was often this feeling that it was terribly uncomfortable. And then they quickly changed the subject.”*

*P4: “I also sometimes get the feeling that during small talk, there’s a deliberate tiptoeing around the topic. It’s like they don’t ask about how my parents are, or allowing the conversation to naturally drift toward my brother. Instead, it seems like there’s a forced shift away from it.”*

*P9: “And then, he was never mentioned again. I always found that was a bit of a pity. He had always been a bit excluded.”*

Participants linked these experiences to the discomfort and uncertainty others felt about discussing grief and suicide in everyday conversations. Furthermore, participants described instances where their loss was insensitively compared to other suicide cases. They felt this sensationalized the death and trivialized their grief by focusing solely on the method of suicide, ignoring the wider context of their loss and pain:

*P3: “One reaction I found really awful was when I talked to someone I met during my internship. She mentioned that an acquaintance of hers, who was otherwise a stranger to her, had also taken her own life, also by train. (…) I was just speechless at first. I didn’t know what to say. (…) So, I found that very, it was really terrible. It troubled me a lot afterward. But then I just thought to myself, ‘Why can’t you stand the fact that the topic is unpleasant for ten minutes, just for ten minutes? That it’s difficult and you have to endure my emotions for a moment?’ So, that actually hurt me a lot.”*

Participants recounted receiving unsolicited and insensitive advice about managing their personal lives and grief. One participant felt pressured by others repeatedly approaching her about her ambitions to find a new partner a few months after the death of her spouse, which she perceived as a profound lack of sensitivity and understanding:

*P10: “[That’s] so tasteless or insensitive. It shows how quickly people, especially those who aren’t close to you, forget that the time for that just hasn’t come yet.”*

Another participant shared how acquaintances inappropriately suggested she sell her house, the site of her son’s death, which she felt was an imposition on her personal choices and a dismissal of her coping with grief:

*P11: “That was also the first reaction from most people, ‘Oh, you have to sell the house. You can’t be happy in that house anymore,’ and so on. Where you get something imposed on you. And then you say, ‘No, this is my house. Yes. Something very, very terrible happened in this house. But we want to continue living here.’”*

Furthermore, participants detailed challenging situations in their workplace where they felt a lack of genuine support from their supervisors, who seemed to expect a predictable grief process from their employees. These interactions were perceived as dismissive and neglectful of SLS’ emotional needs during their bereavement journey:

*P10: “Yes, so my supervisor already told me to take the time that I needed. But when I come back, I should be, well, ready and consistent, just as an employer would like, fit for work again.”*

*P6: “I felt the need to explain to my boss why I was missing work more often. And she had somehow reacted really stupidly. I told her what had happened, that it was suicide. This was about two months later. But she then said that we would need to organize a replacement and that the team would have to coordinate better to make it work. And that was it, basically. It really stuck with me. How cold her reaction was. I’m not even sure if she offered her condolences or anything.”*

## Discussion

### Overview of findings

We examined the social experiences of SLS across three distinct phases and contexts of bereavement: the immediate aftermath of the loss, during bereavement practices, and over time. By exploring both supportive and non-supportive experiences, we have outlined the range of social interactions and encounters following suicide loss and identified factors that either facilitate or impair SLS’ perceptions of social support.

#### Initial support dynamics

During the initial period of suicide bereavement, proactive gestures of acknowledgment, personal condolences, and symbolic acts of sympathy emerged as crucial in conveying social support, validating SLS’ grief and fostering a sense of widespread community support. However, this period also revealed experiences characterized by others’ discomfort or curiosity, including social avoidance and intrusive questions regarding the details and circumstances of the death, intensifying SLS’ distress and feelings of isolation.

#### Mourning practices and social connections

Mourning and commemorative events played an important role in facilitating community support, especially interactions involving the deceased’s social network, which fostered a sense of mutual empathy and shared grief. Personalized mourning practices that included the deceased’s belongings and spaces promoted meaningful social engagement, enhancing SLS’ sense of support and connection with others. In contrast, shock and general feelings of uncertainty among funeral attendees as well as personal discomfort with such events left SLS feeling disconnected. Furthermore, COVID-19 policies on social gatherings restricted support opportunities during these events.

#### Long-term social experiences

Over time, supportive social interactions mainly stemmed from peers and persons who had encountered significant life challenges themselves, offering profound understanding and recognition of SLS' grief. Such interactions facilitated a culture of emotional openness and vulnerability, integrating SLS' experiences into everyday conversations. Conversely, when others marginalized suicide loss through avoidance and superficial engagement, they obstructed meaningful conversations. This made SLS' loss experiences feel depersonalized or de-emotionalized, thereby isolating this crucial aspect of their lives within their social networks.

### Fostering a culture of empathy and recognition

Our findings align with previous research in highlighting the crucial role of proactive and empathetic social responses in the early stages of suicide bereavement [8, 33]. Such support is crucial for conveying solidarity toward SLS, acknowledging their pain and need for compassion. Echoing previous findings, peer interactions emerged as a valued component of social support. These interactions not only foster a sense of belonging but also help reduce stigma, isolation, and self-blame [34]. Connecting with peers gives SLS hope and empowers them to cope with their grief and the changes that result from their loss [34]. Interestingly, our research suggests that the benefits of peer support extend beyond formal groups, as examined in previous studies [34–37], to include informal networks and connections with others who have experienced similar losses or life challenges. These interactions encouraged open and honest communication, expanding the supportive networks of SLS. In a German interview study among female SLS [7], positive disclosure experiences were noted to help SLS build stronger social connections with others. The work of Levi-Belz and colleagues highlights self-disclosure as a crucial mechanism in coping with suicide loss that fosters supportive social interactions [24] and a sense of belongingness [38].

These findings emphasize the importance of creating empathetic social environments that encourage disclosure and emotional openness following suicide loss. Nonetheless, the benefits of disclosure are likely to depend on the nature of the social feedback SLS receive [7]. Our study indicates that the premises and benefits of disclosure are realized when SLS are welcomed into a community that is open to finding common ground and share vulnerabilities.

### Communication challenges, stigma, and cultural norms

Our study shows that, particularly in cases of social avoidance and when others struggled to express sympathy effectively, there was a significant lack of perceived support among SLS. This observation is in line with existing research that points to “social ineptitude” [18] as a barrier to effective communication following suicide loss. The literature identifies several factors that compound communication difficulties in suicide bereavement. These include the absence of clear social norms for responding to suicide loss, resulting in a “norm of respectful silence” [18]; the societal taboo surrounding suicide, which restricts open discussions about and expression of suicide bereavement [9, 17]; and the sudden and shocking nature of suicide, creating uncertainties in how to interact with SLS [9, 18], while also fueling a “morbid fascination” [9] with the subject.

Some participants empathized with the awkwardness and hesitations their social circles faced. However, such responses intensified their sense of public stigma and feelings of isolation. Research from the UK on sudden loss bereavement suggests that these experiences can lead to ‘disenfranchised grief’ [17]– a lack of social recognition for the loss [39]– potentially undermining social support and reinforcing self-stigma among SLS [9]. Notably, shame and self-stigma, along with avoidance behaviors like non-disclosure and withdrawal prevalent among SLS [14], can influence their perceptions of social interactions. However, it is crucial to recognize the cyclic interplay between SLS’ self-views and the social context surrounding suicide loss. How society addresses suicide loss and how communities react to SLS can either exacerbate or alleviate the self-stigma experienced by SLS, highlighting the importance of supportive social experiences early on in guiding the grieving process of SLS.

Furthermore, our findings suggest that cultural norms surrounding grief, perceived as moral “imperatives to mourn in particular ways and at particular times” [40], can act as barriers to support. As shown in our study, these norms can shape expectations around the grief journey and coping mechanisms, leading to challenges at work and unsolicited advice by others. The impact of these norms was also evident during funerals, which, while being settings that encourage public mourning, may not accommodate a bereaved person’s authentic feelings and needs [40]. This mismatch can make community mourning practices more challenging than supportive.

Our research emphasizes the complexity of navigating grief and social support within particular cultural and social contexts and highlights the necessity for a more adaptable understanding of grief that respects the diverse range of emotional and coping responses.

### Redefining mourning practices and fostering empathetic bonds

Funerals and memorials are important settings for collective mourning, offering spaces where communities can together process their loss [1, 41]. Our findings reflect a broader trend of shifting away from traditional religious rituals toward more personalized and secular mourning practices in Western societies [42]. We found that bereavement practices customized to reflect the deceased’s life and relationships greatly enhanced the social support experienced by SLS. This observation aligns with a U.S. study that underlines the benefits of these rituals, particularly through providing emotional safety and personal meaning to the bereaved [43]. Existing research highlights the benefits of positive funeral experiences and post-funeral rituals not only in aiding in the adjustment to loss [41], but also in enhancing connections among those bereaved [43]. The concept of ‘continuing bonds’ [44]– which emphasizes maintaining connections with the deceased throughout a bereaved person’s life– finds practical expression in these personalized mourning practices. While studies in the context of suicide loss are scarce, initial evidence indicates that SLS find value and support in public rituals, enabling the expression of continuing bonds [45]. Research on bereavement after expected deaths has linked elements of continuing bonds to increased feelings of social support [40]. Extending these findings, our research emphasizes the importance of shared mourning through communal practices, especially those engaging the deceased’s social network, in promoting continuing bonds and enhancing social support after suicide loss.

### Limitations

Based on qualitative interviews with SLS in Germany and framed by Western research paradigms, our findings might not fully apply across diverse cultural and social contexts. The limited diversity of our sample, which consists entirely of individuals of German nationality, predominantly non-migrant, and with a high level of education, may not reflect the wide range of experiences shaped by different personal, socio-structural, and cultural factors. Our methods, including purposive sampling and advertising through regional newspapers and SLS support groups, might have introduced a selection bias. Those who chose to participate may be inherently more open to communication, potentially skewing the representation of social experiences toward those of SLS more inclined to disclosure and resilient to stigma. Consequently, our study may not fully represent the experiences of SLS experiencing intense feelings of shame, guilt, and self-stigma, or those encountering additional barriers to receiving support, such as social isolation or health restrictions. Notably, our sample largely comprised individuals who had lost beloved family members, with only one person who had lost a close friend to suicide. This might overlook the experiences of SLS questioning the legitimacy of their grief and support needs due to non-familial relationships or strained relationships with the deceased. This highlights the need for cautious interpretation of our findings and for future research to explore more diverse perspectives on the social experiences associated with suicide bereavement.

### Future directions

Our research identifies key areas to enhance social support for SLS, focusing on overcoming barriers such as stigma, taboo, and grief norms that impede supportive interactions and positive disclosure experiences. The ‘compassionate communities’ approach [46, 47], which is rooted in public health palliative care and utilizes collective compassion as a strategic social asset, offers a valuable framework to promote inclusivity and awareness of suicide bereavement. Building on insights from stigma research related to mental illness [48], public education initiatives aimed at enhancing grief and suicide literacy, along with efforts to encourage social engagement with those affected by suicidality and suicide loss, can play a crucial role in destigmatizing these experiences. Such approaches could empower communities to engage in open and empathetic conversations with SLS.

SLS encounter significant challenges, particularly in coping with stigma and navigating stressful social situations. This emphasizes the need for stigma-sensitive programs that directly support SLS and promote their inclusion into empathetic communities. Peer support groups have emerged as an effective tool in this context, offering a platform for empowerment and mitigating perceived stigma among SLS [36]. Moreover, programs like the ‘Honest, Open, Proud’ (HOP) program [49], designed to assist individuals with mental illness in making decisions about disclosure, offers a promising model for adaptation to meet the needs of SLS [11]. This intervention could address the unique stigma challenges associated with suicide loss, thereby reducing stigma-related stress [49] and providing SLS with strategies to cultivate more supportive social environments.

However, to effectively support SLS and develop impactful interventions, a more comprehensive understanding of SLS’ support systems and preferences is needed. Entilli et al. [50] point out the variability in SLS’ perceptions of social support and help-seeking behaviors, influenced by factors such as gender and previous support experiences. For instance, individuals lacking social support turned to online forums and relied on support from individuals outside their immediate network [50]. These findings suggest the insufficiency of a one-size-fits-all approach to supporting SLS and the need for tailored strategies to meet the varied needs of specific SLS groups. For this, future research must broaden its scope to encompass underrepresented perspectives, including those who have lost friends or grandchildren and individuals from marginalized communities. To define what constitutes “good grief support” [2] in the context of suicide bereavement, future research should continue to examine how different sources of social support (family, friends, and extended networks) cater to various support needs (emotional, instrumental, and appraisal) and their impact on reducing stigma and enhancing health outcomes among SLS. Another critical area for future research involves understanding the attitudes, willingness, and perceived competencies to provide social support within SLS’ networks and among the general public, which is key to reducing barriers and fostering supportive environments.

To our knowledge, this study is the first to explore the benefits of personalized bereavement practices in sustaining social support following suicide loss. Increasing awareness and access to these practices through various channels, including media, funeral services, peer support groups, and mental health professionals, can enable SLS to develop post-funeral rituals that foster shared grief and mutual support within their communities. However, further research is essential to fully understand the effects of such practices on SLS’ perceptions of social support. It is also important to explore how these practices promote positive continuing bonds with the deceased without increasing the potential for exacerbating grief complications [45]. Given the complex nature of suicide bereavement, often characterized by difficulties in making sense of the loss, traumatic memories, and negative thoughts and emotions about the deceased [4, 45], this area warrants further investigation.

Furthermore, the COVID-19 pandemic has significantly disrupted bereavement practices and social support systems [51, 52], emphasizing the need for future research aimed at identifying those most vulnerable to the challenges of the pandemic and evaluating social media support mechanisms, as observed during the COVID-19 pandemic [53]. Recent findings by Entilli et al. [54] on bereavement during the pandemic suggest that online support groups can serve as platforms for survivors to constructively articulate their loss experience and access continuous support, highlighting the potential for online communities to complement conventional offline peer support. Innovative ways, such as virtual memorial services and online support groups, may not only provide valuable community support during times of physical distancing but also suggest a blueprint for more adaptable support mechanisms in decentralized communities.

## Conclusions

This study highlights the perspective that, beyond the support provided by immediate family and friends, diverse social experiences are crucial in shaping perceptions of social support in suicide bereavement. We explored both supportive and non-supportive social experiences among SLS, gaining a comprehensive understanding of how social interactions can serve as valuable support resources, enhancing social recognition and community integration, or conversely, contributing to feelings of isolation and stigmatization.

Our findings show that timely and proactive social engagement, along with open communication and peer connections, are essential for providing SLS with a sense of social support. Key findings highlight the support gained from personalized mourning practices and maintaining connections within the deceased’s social network. Moreover, our research underscores how suicide stigma and the prevalent norms of silence and avoidance act as barriers to social support in suicide bereavement. These insights enrich the existing literature and identify avenues for advancing efforts in both practice and research to improve social support for SLS. Accordingly, this study calls for societal and communicative shifts toward a culture of compassion and inclusion in addressing suicide bereavement. We advocate for multifaceted approaches that combine public education, initiatives based on compassionate community principles, and targeted peer interventions. These efforts should aim to reduce stigma, enhance awareness of the varied experiences of bereavement, and foster environments that encourage open, empathetic conversations about suicide and grief.

## Electronic supplementary material

Below is the link to the electronic supplementary material.

Supplementary material 1: Interview guide section: SLS’ social experiences within their broader social environments

## Data Availability

The datasets generated and analyzed during the current study are not publicly available to protect participants’ privacy and maintain confidentiality but are available from the corresponding author on reasonable request.

## Footnotes

1 In the original sample (n = 20), two individuals had experienced the loss of a close person to assisted suicide. Analysis revealed significant differences between the experiences of SLS and survivors after assisted suicide, particularly in their anticipation of the event and involvement in its planning and completion. These distinct experiences not only presented specific challenges but also influenced social interactions, available support options, and support needs. To ensure comparability, interviews with survivors after assisted suicide were excluded, resulting in a final sample size of 18 SLS. Inspired by these interviews, a narrative literature review exploring the experiences and support needs of individuals bereaved by assisted suicide was conducted and published [30].

## Abbreviations

SLS: Suicide loss survivors
COREQ: Consolidated Criteria for Reporting Qualitative Research
DFG: Deutsche Forschungsgemeinschaft, German Research Foundation
WMA: World Medical Association
