# Supplementary material for: Supportive and non-supportive social experiences following suicide loss: a qualitative study
Source: BMC Public Health. 2024 Apr 27;24:1190. doi: 10.1186/s12889-024-18545-3 (PMC11055309; doi:10.1186/s12889-024-18545-3)
Supplement: Supplementary file 1 — Supplementary material 1: Interview guide section: SLS’ social experiences within their broader social environments [file 12889_2024_18545_MOESM1_ESM.pdf]

Interview guide section: **SLS' social experiences within their broader social environments**

theme	question
narrative prompt	Reflecting on the time following [name of deceased]'s passing, could you share with me how your wider circle, including acquaintances and more distant contacts, responded?
social reactions, interactions, encounters	How did they react upon hearing about [name of deceased]'s passing?<br>• Were most people in your wider circle aware of [name of deceased]'s passing before you told them, or did they find out from you?<br>• Were there any reactions or encounters, positive or negative, that particularly stand out to you?<br>• Were there any interactions or responses that felt particularly hurtful or insensitive to you? Could you describe a specific instance that stands out and how it affected you?<br>• Were there any moments of kindness or support that particularly touched you or brought you comfort? Could you share a specific encounter or gesture that made a significant positive impact on you?<br><br>Can you tell me about the ways you coped with or responded to the range of reactions from your social circles?<br>• How did specific reactions you received impact your feelings and your grieving process?<br>• Were there any aspects you found particularly challenging?<br>• Were there strategies or supports that you found particularly helpful?<br><br>Reflecting on the different reactions you encountered, do you have thoughts on why people reacted the way they did?<br>• Are there any factors you think influenced these reactions?<br><br>*(Follow-up questions about other social contexts, such as workplace, neighborhood, club, friends of the deceased.)*

changes within the social environment	Since [name of deceased]'s passing, have you noticed any shifts in your relationships with people around you?<br>• Could you share how some relationships may have grown stronger or become more distant?<br>• What do you think influenced these changes?<br><br>In your interactions with new acquaintances since the loss, how, if at all, does the topic of your loss come into conversation?<br>• What has been your experience in navigating these conversations?<br><br>Have you observed any differences in reactions or interactions based on whether people were aware of the cause of [name of deceased]'s passing?<br>• How did these differences manifest, and what are your thoughts on why they occurred?
